# Effects of rifampin on the pharmacokinetics and pharmacodynamics of milvexian, a potent, selective, oral small molecule factor XIa inhibitor

**DOI:** 10.1038/s41598-022-25936-2

**Published:** 2022-12-23

**Authors:** Vidya Perera, Zhaoqing Wang, Susan Lubin, Lisa J. Christopher, Wei Chen, Sophia Xu, Dietmar Seiffert, Mary DeSouza, Bindu Murthy

**Affiliations:** Bristol Myers Squibb, 3401 Princeton Pike, Lawrenceville, NJ 08648 USA

**Keywords:** Pharmacology, Cardiovascular diseases

## Abstract

Milvexian (BMS-986177/JNJ-70033093) is a potent, oral small molecule that inhibits the active form of factor XI with high affinity and selectivity. This study assessed the single-dose pharmacokinetic and pharmacodynamic properties of milvexian co-administered with rifampin, an organic anion transport protein (OATP) inhibitor and potent cytochrome P450 (CYP) 3A and P-glycoprotein (P-gp) inducer. In this open-label, nonrandomized, single-sequence study, healthy participants (*N* = 16) received single doses of milvexian on Day 1 (100 mg), milvexian and rifampin (600 mg) on Day 4, rifampin on Days 5–11, milvexian and rifampin on Day 12, and rifampin on Days 13–14. Pharmacokinetic data were summarized using descriptive statistics. Administration of milvexian, alone or in combination with rifampin, was generally safe and well tolerated. Single-dose co-administration of rifampin and milvexian demonstrated no meaningful changes in milvexian exposure versus milvexian alone (C_max_, 110%; AUC_[0–T]_, 102%; AUC_[INF]_, 101%). After multiple doses of rifampin and milvexian, peak and total milvexian exposure substantially decreased versus milvexian alone (C_max_, 22%; AUC_[0–T]_, 15%; AUC_[INF]_, 15%). Results were consistent with preclinical data, indicating that milvexian is a substrate for CYP3A4/5 and P-gp but not OATP. The implications of these results on the need for dose adjustment of milvexian will be further elucidated following the completion of phase 2 and 3 trials.

*Trial registration* The study was registered with ClinicalTrials.gov (NCT02959060; submitted 7/11/2016, first posted 8/11/2016).

## Introduction

The clinical benefits of anticoagulant therapy are well established for the prevention of thrombosis in patients with existing cardiovascular disease. Blood coagulation involves the coordinated activation of plasma proteases, their co-factors, and platelets, with 2 distinct coagulation pathways that converge at factor X^1–3^. Factor XI (FXI) is a component of the intrinsic pathway and has been proposed to play an important role in maintaining and propagating a formed thrombus. Activated factor XI (FXIa) enhances the formation and stability of clots and amplifies thrombin generation when coagulation is initiated by either tissue factor or thrombin^1–3^. Epidemiologic data collected over the past 2 decades indicate that FXIa plays a greater part in thrombosis than in hemostasis^4–7^. Moreover, the attractiveness of FXI as a therapeutic target for anticoagulation is further supported by the lack of spontaneous bleeding associated with FXI deficiency^2,8^. The inhibition of FXI may provide a novel mechanism for systemic anticoagulation with the potential to improve the benefit/risk profile observed with existing anticoagulants through greater efficacy or a safer bleeding profile^3,9^.

Milvexian (BMS-986177/JNJ-70033093) is an oral small molecule that inhibits FXIa with high affinity and selectivity^10^. Milvexian is being developed to prevent thromboembolic events in diverse patient populations. In preclinical models of arterial and venous thrombosis, milvexian demonstrated antithrombotic activity while preserving hemostasis and was generally safe and well tolerated in phase 1 studies in healthy participants and participants with hepatic impairment^11–14^. Renal excretion of milvexian was low (< 20%) in healthy volunteers administered a single-dose oral suspension^13^.

In addition to being cleared by the renal pathway, unpublished preclinical results indicate that milvexian is a substrate of cytochrome P450 (CYP450) 3A4/5 and P-glycoprotein (P-gp). Unpublished in vitro data indicate that milvexian may not be a substrate of organic anion transport protein (OATP), which facilitates the uptake of drugs into the liver.

CYP450 inducers increase drug metabolism and are commonly used in drug-drug interaction (DDI) studies^15^. Rifampin is a strong inducer of CYP3A, signifying that it can cause a ≥ 80% decrease in the area under the curve (AUC) of sensitive substrates (ie, substrates that demonstrate a decrease in AUC of ≥ fivefold with strong inducers)^15^. Additionally, rifampin is a P-gp inducer as well as an inhibitor for OATP1B1 and OATP1B3 and can cause a ≥ twofold increase in AUC when co-administered with an OATP substrate^15–17^. In clinical use, rifampin is an antibiotic with indications in the United States for the treatment of tuberculosis and meningococcal carrier state, and oral daily doses are not to exceed 600 mg/day^18^; similar doses have been used in previous DDI studies^16,19^. Investigation of the DDI of rifampin with milvexian will model a maximum effect of both CYP3A and P-gp induction as well as OATP inhibition.

Understanding the effects of CYP3A induction on the pharmacokinetic (PK) and pharmacodynamic (PD) properties of milvexian will help inform the safety of dose strength in patients who are taking a CYP3A inducer. This study assessed the impact of potent CYP3A and P-gp induction on the PK and PD properties of milvexian following co-administration of milvexian with repeated doses of rifampin and assessed the impact of OATP inhibition by single-dose co-administration of milvexian and rifampin in healthy volunteers.

## Methods

### Ethics

The study was conducted in accordance with Good Clinical Practice, as defined by the International Council for Harmonisation and in accordance with the ethical principles underlying European Union Directive 2001/20/EC and the US Code of Federal Regulations, Title 21, Part 50 (21CFR50), and was conducted in accordance with the ethical principles that have their origin in the Declaration of Helsinki. Prior to the initiation of the study, the protocol, amendments, and participant informed consent received appropriate approval by the Institutional Review Board/Independent Ethics Committee of IntegReview (now Advarra; Columbia, MD, USA), and all participants provided written informed consent, including consent for any screening procedures conducted to establish participant eligibility for the study. The study was registered at ClinicalTrials.gov (NCT02959060; submitted 7/11/2016, first posted 8/11/2016).

### Study design

**Figure 1:**
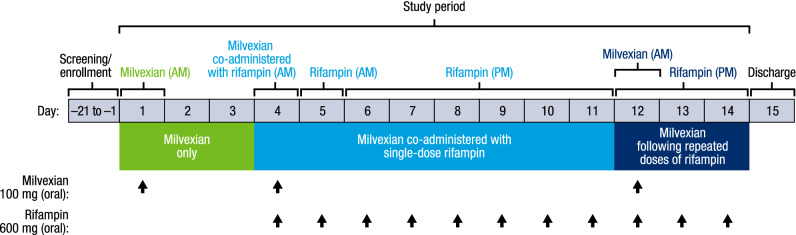
Study design.

The study was an open-label, nonrandomized, single-sequence study in normal healthy participants conducted at 1 clinical research center in the United States. Participants underwent screening evaluations to determine eligibility within 21 days before study drug administration. On the morning of Day 1, participants received a single dose of milvexian 100 mg oral suspension followed by a washout on Days 2 to 3 (milvexian only; Fig. 1). On the morning of Day 4, participants received a single dose of milvexian 100 mg oral suspension co-administered with rifampin 600 mg (2 × 300-mg capsules) followed by daily doses of rifampin 600 mg in the morning on Day 5 and in the evening on Days 6 through 11 (milvexian co-administered with single-dose rifampin). Co-administration of milvexian and rifampin in the morning was used to evaluate the maximum impact of OATP inhibition. On Day 12, participants received a single oral dose of milvexian 100 mg in the morning and daily doses of rifampin 600 mg in the evening on Days 12 to 14 (milvexian following repeated doses of rifampin). Administration of milvexian in the morning and rifampin in the evening was intended to minimize any potential concurrent effects of OATP inhibition when evaluating CYP3A induction. Absorption of rifampin is reduced by approximately 30% when administered with food^18^; therefore, patients in the current study were fasted for 8 h prior until 4 h after study treatment administration on Days 1, 4, and 12 and for 2 h prior until 2 h after study treatment administration on all other days.

When selecting the dose in this study, two key considerations about the potential for induction and inhibition from rifampin were taken into account. Milvexian was not a substrate for OATPs based on pre-clinical knowledge; however, it had shown clear metabolism via CYP3A4/5. Therefore, the potential for an increase in milvexian exposure due to OATP inhibition was considered low while the potential for a decrease in milvexian exposure due to CYP3A4/5 induction was considered high. The first-in-human study showed that doses between 20 and 200 mg were generally dose proportional and single doses up to 500 mg (with food) were safe^13^. Therefore, the spray-dried dispersion (SDD) suspension formulation at 100 mg was selected in this study with the knowledge that there was sufficient safety margin for any potential increases due to OATP inhibition while having sufficient exposures to characterize milvexian following the induction due to CYP3A4/5.

### Participants

The study included healthy participants aged 18 to 55 years with a body mass index of 18.0 to 30.0 kg/m^2^. Women of childbearing potential or who were breastfeeding were excluded. Other exclusion criteria included significant acute or chronic medical illness, or any other condition listed as a contraindication in the rifampin package insert, evidence of coagulopathy, or a history of bleeding.

### Safety assessments

Safety assessments were based on medical review of adverse event (AE) reports and the results of vital sign measurements, electrocardiogram (ECG) measurements, physical examinations, and clinical laboratory tests. Participants were closely monitored throughout the study for nonserious AEs and serious AEs (SAEs).

### Pharmacokinetic and pharmacodynamic assessments

PK assessments included maximum observed plasma concentration (C_max_), area under the plasma concentration–time curve from time 0 to time of the last quantifiable concentration (AUC_[0–T]_), area under the plasma concentration–time curve from time 0 extrapolated to infinite time (AUC_[INF]_), time of maximum observed plasma concentration (T_max_), and terminal plasma half-life (T_1/2_) throughout the study.

Activated partial thromboplastin time (aPTT) and FXI clotting activity were assessed as PD biomarkers. PD parameters were measured at Labcorp Colorado Coagulation (Englewood, CO).

Blood samples were collected for PK analysis immediately prior to dosing and at time points up to 72 h after dosing with milvexian on Days 1, 4, and 12; samples for PD analysis were collected immediately prior to dosing and at time points up to 72 h after dosing on Days 1 and 12 (Supplemental Table S1). Plasma samples were analyzed for milvexian by a validated liquid chromatography tandem mass spectrometry assay using a Waters liquid chromatography system with an AB Sciex mass spectrometer. Briefly, milvexian was extracted by protein precipitation followed by high-performance liquid chromatography separation. Stable labeled milvexian was used as the internal standard. PK parameters were derived from the respective plasma concentration versus time data using a noncompartmental method and Phoenix® WinNonlin® (Certara USA, Inc., Princeton, NJ; version 6.4).

### Statistical analyses

All plasma milvexian PK and PD data were summarized using descriptive statistics. Confidence intervals (CIs) were constructed using a linear mixed-effects model with treatment as a fixed effect and measurements within participants as repeated measures fitted to the log-transformed PK parameters. Plots of individual treatment ratios of PK parameters (C_max_, AUC_[0–T]_, and AUC_[INF]_) combined with geometric least-square mean ratios and corresponding 90% CIs from the statistical analysis model were also provided. PD parameters were presented as mean and standard deviation (SD). All statistical analyses and calculations were performed using SAS® software (SAS Institute, Inc., Cary, North Carolina; version 9.3).

### Ethics approval and consent to participate

The study was conducted in accordance with Good Clinical Practice, as defined by the International Council for Harmonisation and in accordance with the ethical principles underlying European Union Directive 2001/20/EC and the US Code of Federal Regulations, Title 21, Part 50 (21CFR50), and was conducted in accordance with the ethical principles that have their origin in the Declaration of Helsinki. Prior to the initiation of the study, the protocol, amendments, and participant informed consent received appropriate approval by the Institutional Review Board/Independent Ethics Committee of IntegReview (now Advarra; Columbia, MD, USA). Prior to beginning the study, all participants provided written informed consent, including consent for any screening procedures conducted to establish participant eligibility for the study. The study was registered at ClinicalTrials.gov (NCT02959060).

## Results

### Participant characteristics

A total of 16 participants entered the treatment period and completed the study. Baseline characteristics are summarized in Table 1.

**Table 1:** Baseline characteristics.

	Total (*N* = 16)
Male, n (%)	16 (100.0)
**Age, y**	
Median (range)	33.0 (23–51)
**Race, n (%)**^a^	
White	7 (43.8)
Black or African American	8 (50.0)
American Indian or Alaska Native	1 (6.3)
**BMI, kg/m**^**2**^	
Median (range)	26.50 (21.5–29.3)

BMI, body mass index.

^a^Percentages may not sum to 100.0% due to rounding.

### Safety

Administration of milvexian with or without rifampin was generally safe and well tolerated. No deaths, SAEs, or discontinuations due to an AE occurred during this study. Overall, 5 participants (31.3%) reported an AE during the study, 1 (6.3%) after receiving milvexian alone and 4 (25.0%) after receiving milvexian co-administered with rifampin (Table 2). All AEs were considered mild in intensity and resolved by the end of the study. No notable clinical laboratory test, ECG, vital sign, or physical examination results were observed.

**Table 2:** Adverse events.

AE^a^, n (%)	Milvexian only (*n* = 16)	Milvexian co-administered with single-dose rifampin (*n* = 16)	Milvexian following repeated doses of rifampin (*n* = 16)	Total (*N* = 16)
**Any AE**	1 (6.3)	4 (25.0)	0	5 (31.3)
Chromaturia	0	3 (18.8)	0	3 (18.8)
Headache	0	1 (6.3)	0	1 (6.3)
Lip dry	1 (6.3)	0	0	1 (6.3)

AE, adverse event; SAE, serious adverse event.

^a^Includes on-treatment SAEs within 30 days after the last dose and nonserious AEs within 3 days of the last dose.

### Pharmacokinetics

**Figure 2:**
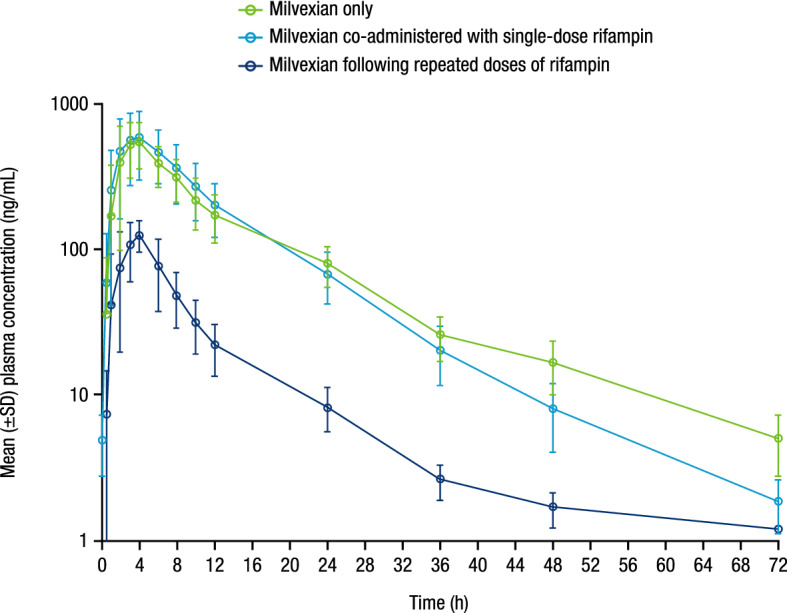
Mean (± SD) milvexian plasma concentration versus time profile. SD, standard deviation.

When milvexian was co-administered with single-dose rifampin, there was no increase in milvexian exposure compared with administration of milvexian alone; however, exposure to milvexian was decreased when administered following repeated doses of rifampin (Fig. 2 and Table 3). Mean T_1/2_ was shorter when milvexian was co-administered with single-dose rifampin and following repeated doses of rifampin compared with milvexian alone (~ 9 vs. 13 h), but median T_max_ was similar across groups (3.5–4 h; Table 3).

**Table 3:** Pharmacokinetic parameters.

	Milvexian only (*n* = 16)	Milvexian co-administered with single-dose rifampin (*n* = 16)	Milvexian following repeated doses of rifampin (*n* = 16)
C_max_, ng/mL	599 (526, 682)	659 (560, 775)	132 (119, 147)
AUC_(0–T)_, ng·h/mL	6153 (5383, 7032)	6293 (5277, 7505)	923 (799, 1067)
AUC_(INF)_, ng·h/mL	6248 (5462, 7148)	6324 (5304, 7541)	958 (837, 1097)
T_max_, h	3.50 (2.00–8.00)	3.54 (1.00–6.13)	4.00 (3.00–6.00)
T_1/2_, h	13.21 (4.075)	8.83 (2.505)	8.85 (2.512)

C_max_, maximum observed plasma concentration; AUC_(0–T)_, area under the plasma concentration–time curve from time 0 to time of last quantifiable concentration; AUC_(INF)_, area under the concentration–time curve from time 0 extrapolated to infinite time; T_max_, time of maximum observed plasma concentration; T_1/2_, terminal plasma half-life; CI, confidence interval; SD, standard deviation.

C_max_, AUC_(0–T)_, and AUC_(INF)_ are presented as adjusted geometric mean (90% CI).

T_max_ is presented as median (minimum–maximum).

T_1/2_ is presented as mean (SD).

**Figure 3:**
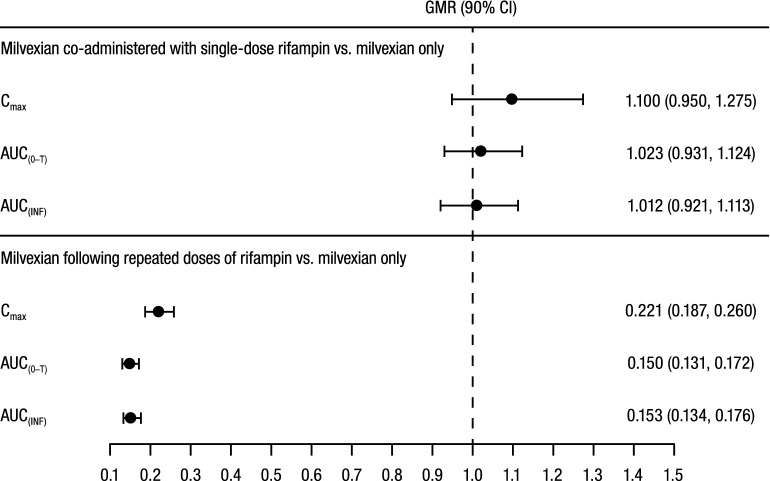
Effect of rifampin on milvexian PK parameters. PK, pharmacokinetic; GMR, geometric mean ratio; CI, confidence interval; C_max_, maximum observed plasma concentration; AUC_(0–T)_, area under the plasma concentration–time curve from time 0 to time of last quantifiable concentration; AUC_(INF)_, area under the concentration–time curve from time 0 extrapolated to infinite time.

When milvexian was co-administered with single-dose rifampin, C_max_, AUC_(0–T)_, and AUC_(INF)_ were similar to the values observed with administration of milvexian alone (Fig. 3). When milvexian was administered following repeated doses of rifampin, peak and total milvexian exposures were substantially decreased: C_max_, AUC_(0–T)_, and AUC_(INF)_ values were 22%, 15%, and 15% of the exposures observed when milvexian was administered alone (Fig. 3).

### Pharmacodynamics

**Figure 4:**
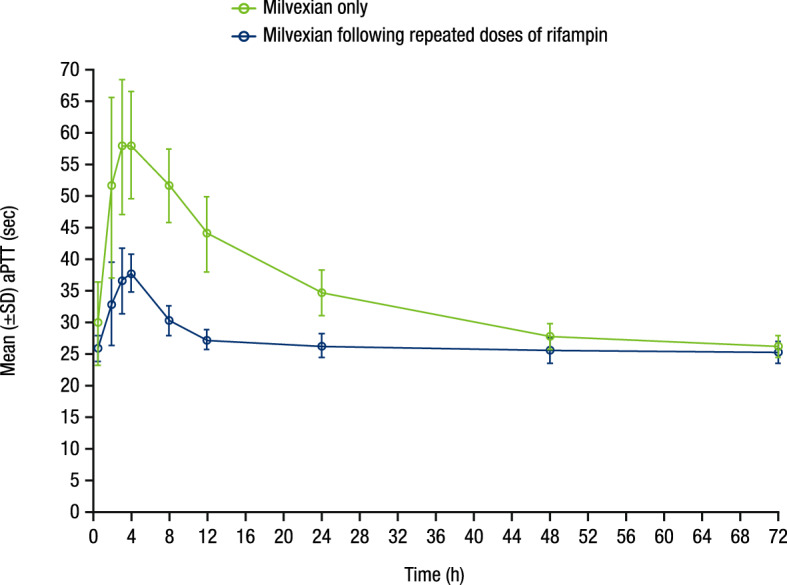
Mean (± SD) aPTT profile versus time profile. SD, standard deviation; aPTT, activated partial thromboplastin time.

Prolongation of aPTT was observed with milvexian administration, with the greatest effects observed around 4 h post-dose (Fig. 4), corresponding with T_max_. The magnitude of change from baseline in aPTT was smaller when milvexian was administered following repeated doses of rifampin than when milvexian was administered alone.

**Figure 5:**
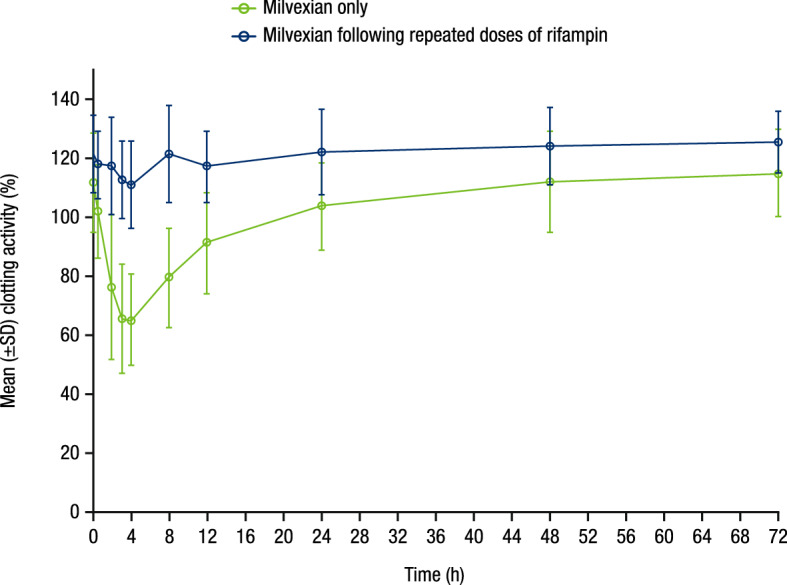
Mean (± SD) FXI clotting activity versus time profile. SD, standard deviation; FXI, factor XI.

A reduction in FXI clotting activity was observed with milvexian administration across all treatment periods, with greater effects observed around 4 h post-dose (Fig. 5), corresponding with T_max_. The magnitude of reduction in FXI clotting activity across the sampling interval was smaller when milvexian was administered following repeated daily dosing of rifampin compared with administration of milvexian alone.

## Discussion

This study assessed the effects of administration of milvexian with rifampin. Rifampin possesses a complex pharmacological profile based on its effects on the nuclear pregnane X receptor (PXR) and constitutive androstane receptor (CAR). It has previously been established that a single acute oral administration of rifampin results in potent inhibition of hepatic OATP1B uptake transporters, while chronic dosing results in induction of CYP3A, UDP-glucuronosyltransferase 1A (UGT1A), P-gp, and multidrug resistance-associated protein 2 (MRP2)^21^. Therefore, this study was designed to elucidate the potential impact of OATP inhibition on milvexian PK parameters by assessment following a single dose of rifampin in the morning of Day 5, with sufficient time to collect PK data, and prior to investigating the impact on CYP3A induction following chronic dosing of rifampin given on the evenings of Day 6 through Day 11. Co-administration led to a maximal effect of rifampin on OATP inhibition. The induction interaction of CYP3A and P-gp was assessed after a morning dose of milvexian, which was followed by administration of daily doses of rifampin in the evenings over multiple days; the strategy of separating the milvexian and rifampin doses was used to minimize the effect of OATP inhibition by rifampin.

When milvexian 100 mg was administered alone, most PK parameters and PD biomarkers were within the range of anticipated results based on the phase 1 first-in-human study in healthy participants, though the results are not directly comparable since a 100 mg dose of the SDD suspension formulation was not investigated in that study^13^. Single-dose co-administration of milvexian and rifampin did not meaningfully change the peak (C_max_) and total (AUC) exposures of milvexian compared with administration of milvexian alone. The T_max_ of milvexian was not altered by co-administration with a single dose of rifampin, but T_1/2_ was shorter. These results indicate that milvexian is not a sensitive OATP substrate.

Administration of milvexian following multiple daily doses of rifampin (resulting in CYP3A and P-gp induction) substantially reduced both peak (C_max_) and total (AUC) exposure of milvexian to between 15% and 22% of the exposures observed after administration of milvexian alone. The reduction in C_max_ may be indicative of a high level of induction of CYP3A and P-gp in the gut, leading to a limited systemic absorption of milvexian. The T_max_ of milvexian was not altered following repeated doses of rifampin; however, T_1/2_ was shorter. These results suggest that milvexian is a substrate for CYP3A4/5 and P-gp. The implications of these results on the need for dose adjustment of milvexian will be further elucidated following the completion of phase 2 and 3 trials.

Consistent with the mechanism of action of milvexian, exploratory assessments of PD biomarkers showed prolongation of aPTT and reduction of FXI clotting activity with milvexian administration, with greater effects observed at higher milvexian concentrations. These exposure-dependent relationships between aPTT or FXI clotting activity and milvexian concentrations were observed irrespective of the presence of rifampin. The magnitude of changes of both PD biomarkers was smaller following repeated doses of rifampin than when milvexian was administered alone, consistent with the lower plasma concentration of milvexian observed following repeated doses of rifampin.

Administration of milvexian 100 mg alone and in combination with rifampin 600 mg was generally safe and well tolerated by the healthy participants in this study. There were no deaths or SAEs, and no participant discontinued the study due to an AE. All AEs were considered mild in intensity and resolved by the end of the study.

A limitation of this study is that data were obtained from a small sample of healthy participants who were all male; however, the conclusions remain robust. The inability to dissociate the impact of P-gp and CYP3A contributions to the induction effect warrants further assessment. At the time of writing this manuscript, the results of the milvexian exposure–response analysis from phase 2 studies and the start of phase 3 is underway; therefore, a conclusion regarding the implications of these results on dosing is not provided. A comprehensive population PK and PK/PD modeling manuscript incorporating covariates including DDIs in addition to other intrinsic and extrinsic factors will further inform the overall dosing recommendations of milvexian.

## Conclusions

The data from this study indicate that milvexian is a substrate for CYP3A and P-gp and is not a substrate for OATP. The implications of these results on the need for dose adjustment of milvexian will be further elucidated following the completion of phase 2 and 3 trials. Milvexian was generally safe and well tolerated in healthy participants. The results of this study will help to inform the future clinical development of milvexian.

## Supplementary Information

Supplementary Information.

## Data Availability

The data that support the findings of this study are not publicly available due to privacy or ethical restrictions. Please contact the corresponding author, Vidya Perera, for additional information.
