# Supplementary material for: Effects of rifampin on the pharmacokinetics and pharmacodynamics of milvexian, a potent, selective, oral small molecule factor XIa inhibitor
Source: Sci Rep. 2022 Dec 23;12:22239. doi: 10.1038/s41598-022-25936-2 (PMC9789074; doi:10.1038/s41598-022-25936-2)
Supplement: Supplementary file 1 — Supplementary Information. [file 41598_2022_25936_MOESM1_ESM.pdf]

Supplementary Information

Effects of rifampin on the pharmacokinetics and pharmacodynamics of milvexian, a potent, selective, oral small molecule factor XIa inhibitor

Vidya Perera,[1,*] Zhaoqing Wang,[1] Susan Lubin,[1] Lisa J. Christopher,[1] Wei Chen,[1] Sophia Xu,[1] Dietmar Seiffert,[1] Mary DeSouza,[1] Bindu Murthy[1]

[1]Bristol Myers Squibb, Princeton, NJ, USA.

Supplemental Table S1. PK and PD sampling schedule

Study day	Time (relative to milvexian dose); hour:min	PK blood sample	PD blood sample
1	00:00	X	X
1	00:30	X	X
1	1:00	X	
1	2:00	X	X
1	3:00	X	X
1	4:00	X	X
1	6:00	X	
1	8:00	X	X
1	10:00	X	
1	12:00	X	X
2	24:00	X	X
2	36:00	X	
3	48:00	X	X
4	00:00	X	X
4	00:30	X	
4	1:00	X	
4	2:00	X	
4	3:00	X	
4	4:00	X	
4	6:00	X	
4	8:00	X	
4	10:00	X	
4	12:00	X	

5	24:00	X	
5	36:00	X	
6	48:00	X	
7	72:00	X	
12	00:00	X	X
12	00:30	X	X
12	1:00	X	
12	2:00	X	X
12	3:00	X	X
12	4:00	X	X
12	6:00	X	
12	8:00	X	X
12	10:00	X	
12	12:00	X	X
13	24:00	X	X
13	36:00	X	
14	48:00	X	X
15	72:00	X	X

PK, pharmacokinetic; PD, pharmacodynamic.
